# Purple Urine Bag Syndrome as the Primary Presenting Feature of a Urinary Tract Infection

**DOI:** 10.7759/cureus.23970

**Published:** 2022-04-09

**Authors:** Michael Popoola, Marston Hillier

**Affiliations:** 1 Internal Medicine, Dorset County Hospital NHS Trust, Dorchester, GBR

**Keywords:** case report, rare, infection, purple urine bag syndrome, delirium, urinary catheter, uti

## Abstract

Purple urine bag syndrome (PUBS) is a curious condition that manifests with purple discoloration of a urinary catheter bag as a result of a urinary tract infection (UTI). This syndrome is produced through a reaction between urinary by-products and the components of a plastic urinary catheter bag. Predisposing factors include old age, long-term catheterization, chronic constipation, and limited mobility. We describe a case of a UTI in a 90-year-old woman in which the initial presentation was PUBS. Here, late recognition of PUBS led to delayed treatment of the associated UTI. Despite being a simple spot diagnosis, knowledge about this uncommon syndrome is lacking amongst clinicians and this may have implications on patient outcomes.

## Introduction

Purple urine bag syndrome (PUBS) is a clinical sign that presents uniquely as a purple-colored discoloration of a urinary catheter bag. This phenomenon was originally described in 1978 [1] and occurs predominantly in elderly, constipated women who are chronically catheterized and have a co-existing urinary tract infection (UTI) [2]. The purple tint arises from a chemical reaction between two urinary substrates (indigo and indirubin) and the synthetic constituents of a polyvinyl chloride urinary catheter bag [3]. The formation of indigo and indirubin arises specifically in the setting of urinary bacteria containing the sulfatase and phosphatase enzymes [4]. Whilst PUBS is often considered a relatively benign process, its development has been associated with increased morbidity and mortality [5-6].

## Case presentation

**Figure 1:**
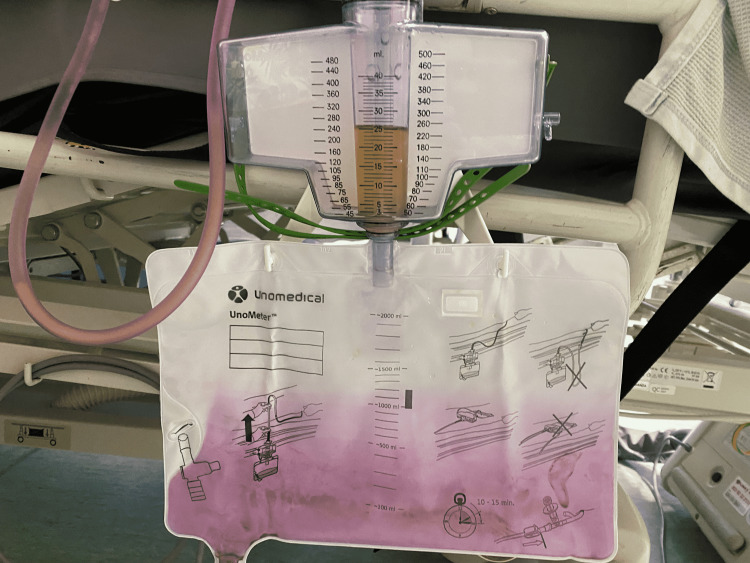
Purple-stained urinary catheter bag.

A 90-year-old woman was admitted with shortness of breath, wheezing, and worsening peripheral edema on a background of heart failure. She was diagnosed with acute decompensated heart failure secondary to myocardial infarction and was managed medically. During her prolonged inpatient stay, the patient developed urinary retention secondary to constipation. She was catheterized for symptomatic relief. She then remained an inpatient for several weeks whilst awaiting a complex discharge plan from the hospital. After approximately one month with catheter in situ, it was identified that her urinary catheter bag had turned purple (Figure 1). The urine itself within the bag was a concentrated brown color.

**Figure 2:**
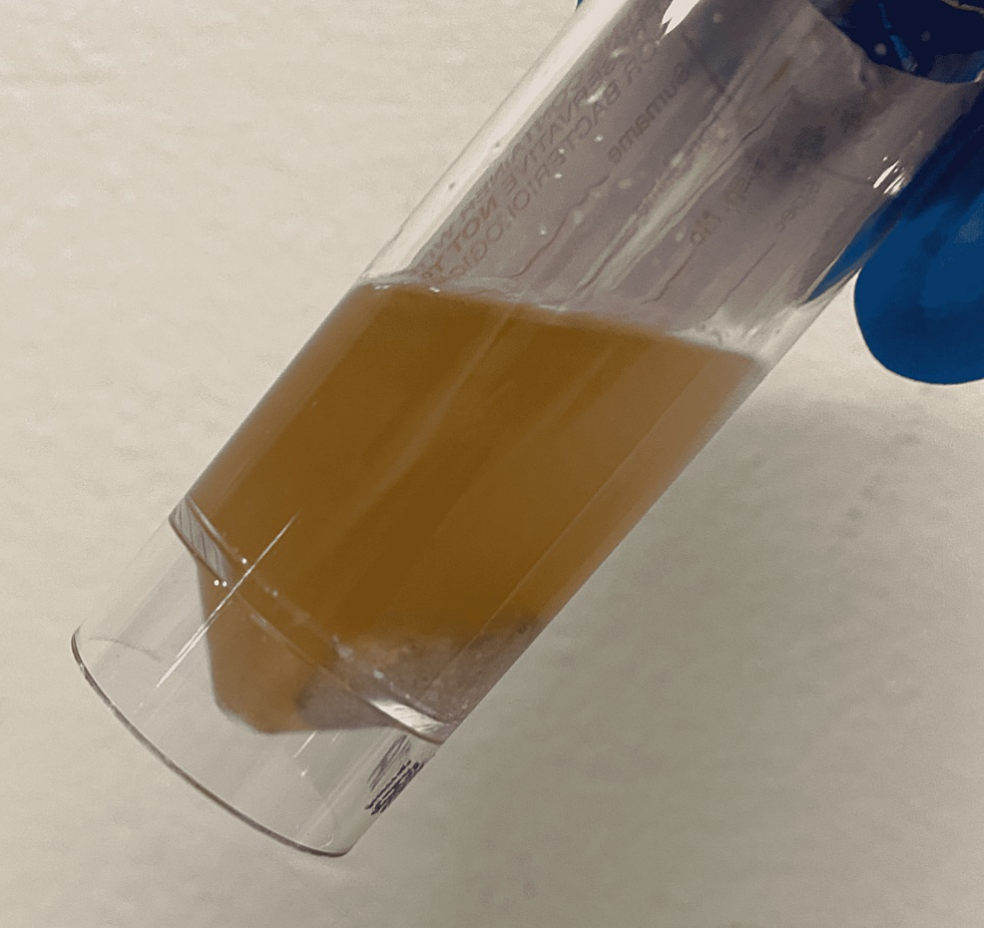
Urine sample from the catheter bag.

The patient denied any classical urinary tract symptoms, specifically: dysuria, suprapubic pain, or urgency, though she had a background of mild cognitive impairment. There were no catheter-associated complications such as overflow or leaking. However, clinical examination revealed marked suprapubic tenderness. Her chart was reviewed and there had been no recent changes to medication. A urine sample, with noticeable sediment/pus (Figure 2), was sent for microscopy, culture, and sensitivity (MCS). The catheter was removed. Blood tests revealed elevated inflammatory markers with C-reactive protein of 57 mg/L (normal 0-5 mg/L) and white cell count of 14.5 x 109/L (normal 4-10 x 109/L) and she was treated for a UTI with empirical antibiotics (ciprofloxacin). MCS later revealed Proteus species with a white cell count of 273 x 106/L (normal 0-40 x 106/L). The patient responded well clinically, had a meaningful improvement in her cognitive status, and was discharged from the hospital about 24 h later.

## Discussion

**Figure 3:**
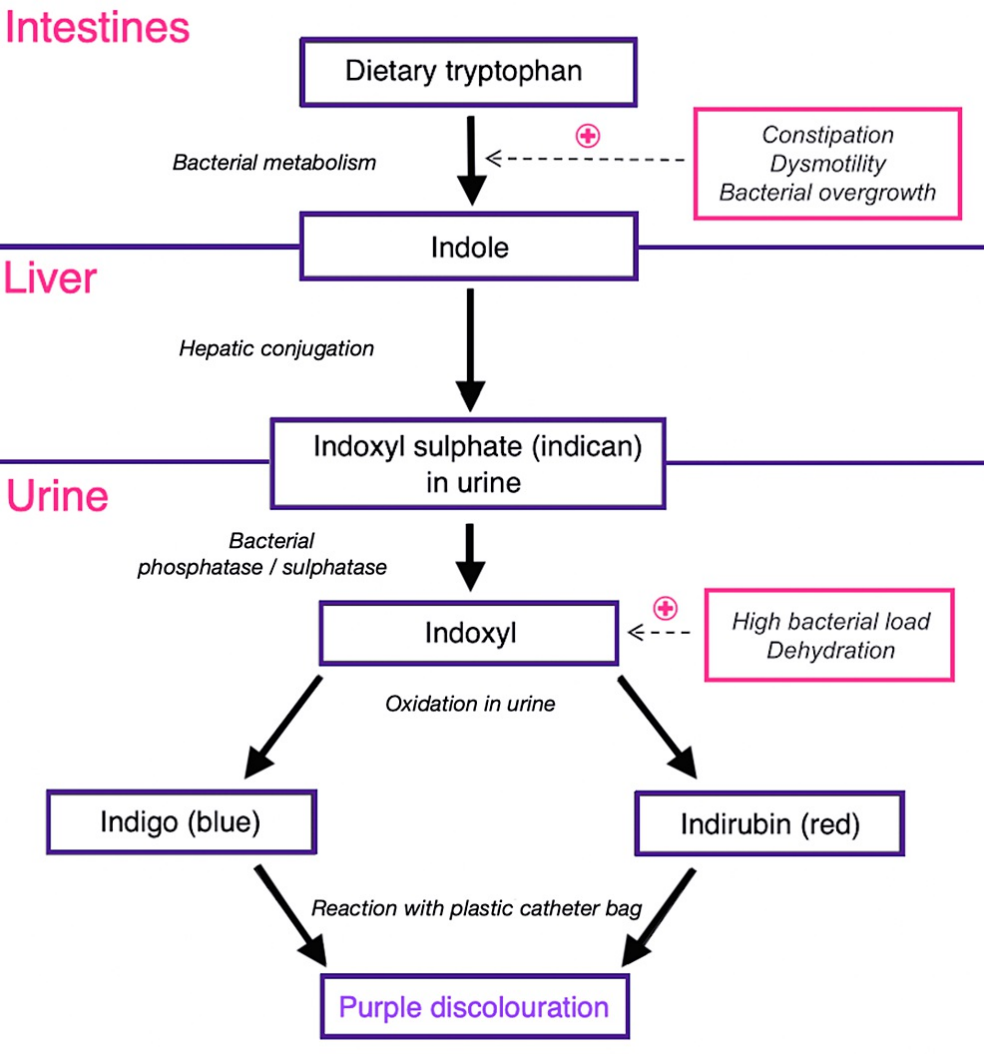
Etiology of purple urine bag syndrome. Adapted from the diagram originally published by Harun et al. [7].

Differential diagnoses for discoloration of urine vary from iatrogenic causes such as medication (rifampicin, methylene blue, IV hydroxocobalamin) to inherited conditions such as alkaptonuria or porphyria. In elderly patients, in the absence of known inherited diagnoses, medication changes or PUBS are much more likely causes of urine discoloration. Here we describe a case of a common medical condition (UTI) in which the initial presenting symptom/sign was the unusual reaction known as PUBS. The pathway leading to this event requires various conditions to be present together; these conditions functionally increase the amount of bacterial metabolism of dietary tryptophans and associated renal clearance of metabolites (Figure 3).

There are several known risk factors for the development of PUBS [2], of which our patient had several: the use of a polyvinyl chloride catheter bag; long-term catheterization; poor mobility; constipation; and increasing age. It was unclear how long the catheter bag had harbored the purple stain in our patient, but from the pattern seen in Figure 1, it was likely at a minimum from the previous day. This could have represented an opportunity for earlier intervention for an ostensibly symptomless UTI in an elderly, frail patient. 

The PUBS itself is an uncommon clinical finding, with fewer than 150 cases described in the literature since 1980 [8]. There is an overall mortality rate of approximately 5% for the described cases [8]. This highlights that the risk factors for the development of PUBS significantly overlap with frailty syndromes. This population of patients have poor outcomes from the hospital-associated catheter-associated UTIs. Increasing awareness of this spot diagnosis may, without the need for time consuming or invasive testing, lead to earlier treatment of UTIs, reduction in inpatient hospital days, and a subsequent decrease in inpatient mortality. 

## Conclusions

The PUBS is an uncommon presentation of a UTI that may be seen in patients with the requisite risk factors. In the patient demographic in which this syndrome predominates, classical UTI symptoms can be difficult to elicit and non-specific presentations, such as delirium, are more commonly seen. PUBS is a straightforward spot diagnosis and thus represents an opportunity to recognize and treat a common medical ailment. Greater awareness of this condition is required amongst clinicians to intervene earlier and avoid misdiagnosis.

## References

[1] Barlow GB, Dickson JAS (1978). Purple urine bags. Lancet.

[2] Su FH, Chung SY, Chen MH (2005). Case analysis of purple urine-bag syndrome at a long-term care service in a community hospital. Chang Gung Med J.

[3] Kalsi DS, Ward J, Lee R, Handa A (2017). Purple urine bag syndrome: a rare spot diagnosis. Dis Markers.

[4] Khan F, Chaudhry MA, Qureshi N, Cowley B (2011). Purple urine bag syndrome: an alarming hue? A brief review of the literature. Int J Nephrol.

[5] Tasi YM, Huang MS, Yang CJ, Yeh SM, Liu CC (2009). Purple urine bag syndrome, not always a benign process. Am J Emerg Med.

[6] Bhattarai M, Bin Mukhtar H, Davis TW, Silodia A, Nepal H (2013). Purple urine bag syndrome may not be benign: a case report and brief review of the literature. Case Rep Infect Dis.

[7] Harun NS, Nainar SK, Chong VH (2007). Purple urine bag syndrome: a rare and interesting phenomenon. South Med J.

[8] Yang HW, Su YJ (2018). Trends in the epidemiology of purple urine bag syndrome: a systematic review. Biomed Rep.

